# Recent trends in the management of advanced prostate cancer

**DOI:** 10.12688/f1000research.15382.1

**Published:** 2018-09-21

**Authors:** Chad Ritch, Michael Cookson

**Affiliations:** 1 Department of Urology, Miller School of Medicine, University of Miami, Miami, FL, USA; 2 Department of Urology, University of Oklahoma College of Medicine and Stephenson Cancer Center, Oklahoma City, OK, USA

**Keywords:** prostate cancer, chemohormonal therapy, castration resistant prostate cancer, hormone sensitive metastatic prostate cancer

## Abstract

Advanced prostate cancer includes a wide spectrum of disease ranging from hormone naïve or hormone sensitive to castration resistant, both containing populations of men who have demonstrable metastatic and non-metastatic states. The mainstay of treatment for metastatic hormone-sensitive prostate cancer is androgen deprivation therapy (ADT). However, recent level 1 evidence demonstrates that the addition of chemotherapy or abiraterone acetate to ADT results in significant survival advantage as compared with ADT alone. Furthermore, in non-metastatic castration-resistant prostate cancer (M0 CRPC), two second-generation anti-androgens, apalutamide and enzalutamide, when used in combination with ADT, have demonstrated a significant benefit in metastasis-free survival. Here, we review the most recent studies leading to these significant changes in the treatment of advanced prostate cancer.

## Introduction

There have been significant strides in the management of prostate cancer over the past decade. The majority of newly diagnosed cases (80%) are localized prostate cancer, and the remaining cases are advanced or metastatic disease ^1^. Overall survival (OS) rates in localized disease are very high; however, this decreases dramatically for advanced and metastatic cases and ranges from 26% to 30% at 5 years ^2^. Unique to prostate cancer is the fact that cancer cells are highly sensitive to the manipulation of the androgen pathway ^3, 4^. Testosterone and its metabolites have a stimulatory effect on prostate cancer cell growth, and hormonal manipulation and castration can induce prostate cancer cell death ^5^. Therefore, the initial management of metastatic prostate cancer is based on androgen deprivation to achieve castrate levels (<50 ng/dL) of circulating testosterone, thereby depriving the cells of their primary fuel for growth.

For decades, androgen deprivation therapy (ADT), via medical or surgical castration, has been the primary treatment of metastatic prostate cancer. However, patients ultimately progress to castration resistance, wherein prostate cancer cells become resistant to ADT and develop mechanisms to proliferate despite castrate levels of testosterone. Patients who progress to castration-resistant prostate cancer (CRPC) progress rapidly and may die within 2 to 4 years ^6, 7^. However, prior to 2004, there were no US Food and Drug Administration (FDA)-approved therapies for CRPC until several landmark randomized controlled trials (RCTs) (TAX-327 and SWOG 9916) demonstrated that patients with metastatic CRPC (mCRPC) treated with docetaxel chemotherapy achieved a significant survival advantage compared with placebo ^8, 9^. Recently, two landmark studies (STAMPEDE and CHAARTED) examined the role of combined chemotherapy and ADT (chemohormonal therapy) as compared with ADT alone in hormone-sensitive disease ^10, 11^. Additionally, the LATITUDE trial and abiraterone arm of the STAMPEDE trial both demonstrated a survival benefit with abiraterone acetate plus prednisone when combined with ADT over ADT alone for hormone-sensitive prostate cancer ^12, 13^. All of these studies demonstrated a statistically significant benefit in OS and have changed the management paradigm in metastatic prostate cancer. In the castration-resistant setting, since 2010 and almost every year thereafter, several key RCTs have demonstrated survival benefit with new therapies before and after docetaxel-based chemotherapy. The culmination of these studies has led to the FDA approval of six new agents, which have varying mechanisms of action, in the management of metastatic and non-metastatic (M0) CRPC: sipuleucel-T, abiraterone acetate, enzalutamide, cabazitaxel, radium-223, and apalutamide ^14– 21^. Of particular significance is the recent approval of apalutamide and enzalutamide in the treatment of M0 CRPC, which was based upon two RCTs (SPARTAN and PROSPER) demonstrating significant improvement in metastasis-free survival (MFS) ^21, 22^. Prior to these trials, there were no approved agents for M0 CRPC.

The purpose of the present review is to provide an overview of the recent trends and advances in the management of metastatic castration-sensitive prostate cancer (CSPC) and M0 CRPC ( Table 1). We will review the literature supporting the approval of upfront chemotherapy in metastatic CSPC as well as recent landmark studies supporting newer therapies for M0 CRPC.

**Table 1.** Summary of recent trials in castration-sensitive prostate cancer and non-metastatic (M0) castration-resistant prostate cancer.

Trial	Year	Agent	Population	Primary endpoint	Outcome summary
CHAARTED	2015	Docetaxel	Castration-sensitive prostate cancer (CSPC)	Overall survival (OS)	13.6-month OS advantage
STAMPEDE	2016	Docetaxel	CSPC	OS	15.6-month OS advantage
LATITUDE	2017	Abiraterone	CSPC	OS	7% 3-year OS advantage
STAMPEDE	2017	Abiraterone	CSPC	OS	17% 3-year OS advantage
SPARTAN	2018	Apalutamide	M0 castration-resistant prostate cancer (M0 CRPC)	Metastasis-free survival (MFS)	24.3-month MFS benefit
PROSPER	2018	Enzalutamide	M0 CRPC	MFS	21.9-month MFS benefit

## Chemotherapy for metastatic castration-sensitive prostate cancer

Historically, following progression to CRPC, docetaxel chemotherapy was the first-line agent based on results of the TAX-327 and SWOG 9916 trials ^8, 9^. TAX-327 demonstrated that docetaxel every 3 weeks significantly decreased risk of death—hazard ratio (HR) 0.76, 95% confidence interval (CI) 0.62 to 0.94, *p* = 0.009—compared with mitoxantrone ^8^. The median survival was 18.9 versus 16.4 months for docetaxel compared with mitoxantrone. In SWOG 9916, docetaxel plus estramustine was compared with mitoxantrone plus prednisone, and there was a 20% reduction in the risk of death with a median survival improvement of about 2 months, favoring docetaxel ( *p* = 0.02) ^9^. Therefore, docetaxel use was limited to the castration-resistant setting. The GETUG-AFU 15 trial was one of the first to investigate the use of docetaxel in the hormone-sensitive setting—about half of the study participants were classified as having low-volume disease—and failed to meet the primary endpoint of OS benefit ^23^. However, further investigations into the role of chemotherapy in hormone-sensitive prostate cancer were conducted in the STAMPEDE and CHAARTED (ECOG 3805) trials ^10, 11^. In the CHAARTED trial, Sweeney *et al*. performed an RCT of docetaxel (six cycles) plus ADT (chemohormonal therapy) compared with ADT alone in 790 men. The trial demonstrated a significantly longer median OS in the chemohormonal arm compared with ADT alone (57.6 versus 44.0 months; HR 0.61, 95% CI 0.47 to 0.81, *p* <0.001) ^10^. Of particular importance was the 17-month survival advantage noted in a subset of patients with high-volume disease (that is, visceral metastases or at least four bone lesions with at least one beyond the vertebral bodies and pelvis) ^10^. Therefore, the benefit of chemotherapy in the hormone-sensitive state appears more pronounced in men with high-volume disease.

In the STAMPEDE trial, James *et al*. demonstrated improved survival in men who received docetaxel at the time of long-term ADT initiation ^11^. Unique to the STAMPEDE trial is the multi-arm, multi-stage design, wherein patients initiating long-term ADT for newly diagnosed metastatic or locally advanced CSPC or high-risk recurrent prostate cancer are randomly assigned to several additional therapies. For men with metastatic disease at the time of random assignment, the docetaxel arm (n = 592) demonstrated a survival advantage (HR 0.76, 95% CI 0.62 to 0.92, *p* = 0.005) with a median survival difference of 15 months (60 months for docetaxel versus 45 months for ADT). The 5-year survival was 50% in the docetaxel arm compared with 39% for the ADT-alone arm ^11^.

## Abiraterone for metastatic castration-sensitive prostate cancer

The implications of the STAMPEDE and CHAARTED trials are significant because chemohormonal therapy has now become a widely considered first-line therapy in high-volume metastatic HSPC. However, the recent LATITUDE trial, which examined the role of abiraterone acetate plus prednisone in combination with ADT in the hormone-sensitive metastatic prostate cancer setting, has also demonstrated a survival advantage compared with ADT plus placebo ^12^. Fizazi *et al*. randomly assigned 1,199 patients with metastatic HSPC to abiraterone acetate plus prednisone combined with ADT versus ADT plus placebo and demonstrated a significant benefit in survival in the abiraterone arm (HR 0.62, 95% CI 0.51 to 0.76, *p* <0.001) ^12^. Furthermore, there was a significant benefit with abiraterone with respect to time to initiation of chemotherapy as second-line therapy following disease progression, although fewer patients received second-line chemotherapy than expected ^12^. James *et al*. studied the abiraterone arm of the STAMPEDE trial, in which 1,917 men were randomly assigned to abiraterone plus ADT compared with ADT alone ^13^. There was a significant 3-year survival advantage for men in the abiraterone arm of 83% versus 76% in the ADT-alone group (HR 0.63, 95% CI 0.52 to 0.76, *p* <0.001) ^13^. Importantly, owing to the multi-arm, multi-stage trial design, the patient population differed slightly from that of the LATITUDE group in that some men had node-positive only disease as well as node-negative, non-metastatic disease. Both trials demonstrated a survival benefit, and, as a result, abiraterone acetate as well as chemotherapy may also be considered in metastatic CSPC. A recent analysis of data from the STAMPEDE trial compared abiraterone with docetaxel in the castration-sensitive state and found no difference in overall and prostate cancer-specific survival ^24^. Therefore, the approval and utilization of these therapies in combination with ADT in the hormone-sensitive state represent a major advance and paradigm shift in the management of metastatic prostate cancer.

## Treatment of non-metastatic castration-resistant prostate cancer

Progression from the hormone-sensitive to the castration-resistant state is defined as a rising prostate-specific antigen (PSA) with T levels below 50 ng/mL. In the clinical trial setting, the Prostate Cancer Clinical Trials Working Group 3 definition of PSA progression is an at least 25% increase, and an absolute increase of at least 2 ng/mL from the nadir PSA, confirmed at least 3 weeks later ^25^. Despite progression to castration resistance, a subset of patients may not harbor detectable metastasis by the traditional imaging techniques used in the trials and therefore are categorized as having M0 CRPC. Patients with M0 CRPC are at high risk for progression to metastatic disease. In fact, within 2 years, about 15% to 33% can develop metastasis, implying that castration resistance may lead to rapid progression and potentially a high risk of mortality in this population ^26, 27^. Until 2018, there were no approved agents for first-line treatment of asymptomatic M0 CRPC. Prior to the current year, guideline recommendations supported continued ADT in M0 CRPC, with close surveillance, because the androgen receptor remains functionally active in this disease state ^28^. There were no level 1 data showing a significant therapeutic advantage with any particular therapy in M0 CRPC. Therefore, this disease state represented a challenging clinical conundrum because although castration resistance is a harbinger of metastatic disease and mortality, there were no efficacious options for treatment. If the patient or treating physician wished to pursue treatment, the first-generation anti-androgens (flutamide, bicalutamide, and nilutamide) and first-generation androgen synthesis inhibitors (ketoconazole with steroid) were occasionally used with variable and limited efficacy ^28, 29^.

In 2016, the STRIVE study demonstrated potential therapeutic benefit with enzalutamide in M0 CRPC. Penson *et al*. randomly assigned 396 men on ADT, with M0 (n = 139) or metastatic (n = 257) CRPC, to enzalutamide (160 mg/day) or bicalutamide (50 mg/day) ^30^. Enzalutamide reduced the risk of progression or death by 76% compared with bicalutamide (HR 0.24, 95% CI 0.18 to 0.32, *p* <0.001) and the median progression-free survival was 13.7 months longer for men in the enzalutamide arm ^30^. Although these findings were noteworthy, they were not sufficient to lead to FDA approval in M0 CRPC.

Recently, apalutamide, a novel non-steroidal anti-androgen which acts as an androgen receptor inhibitor, was studied in patients with M0 CRPC ^21^. The SPARTAN trial randomly assigned 1,207 men with M0 CRPC and a PSA doubling time of less than 10 months to apalutamide versus placebo with a primary endpoint of MFS. Smith *et al*. demonstrated that men in the apalutamide arm experienced a longer time to progression (HR 0.45, 95% CI 0.32 to 0.63, *p* <0.001) and improved median MFS (40.5 months apalutamide versus 16.2 months placebo: HR 0.28, 95% CI 0.23 to 0.35, *p* <0.001) ^21^. Based on the findings of this trial, apalutamide became the first FDA-approved agent in M0 CRPC. The PROSPER trial randomly assigned 1,401 men with M0 CRPC, PSA doubling time of less than 10 months, and PSA of more than 2 ng/mL to enzalutamide versus placebo ^22^. As in the SPARTAN trial, the primary endpoint was MFS. Hussain *et al*. demonstrated that enzalutamide significantly prolonged median MFS (36.6 versus 14.7 months, *p* <0.0001) as well as time to first use of new anti-neoplastic therapy (39.6 versus 17.7 months, *p* <0.0001) and time to PSA progression (37.2 versus 3.9 months, *p* <0.0001) compared with placebo ^31^. Enzalutamide was subsequently FDA-approved for use in M0 CRPC. The importance of patient selection cannot be underestimated. Patients in both of these landmark trials of M0 CRPC were selected on the basis of high risk for metastases as indicated by a rapid PSA doubling time. Furthermore, the newer positron emission tomography imaging-based tracers were not used in these studies and it is possible that a subset of these men had metastases that were not detectable by the limits of conventional computed tomography imaging and nuclear bone scan. In patients with a slow doubling time, observation may be an appropriate management strategy.

## Emerging treatments

Poly(adenosine diphosphate-ribose) polymerase (PARP) is involved in DNA repair, and recent studies have demonstrated an 11.8% incidence of germline mutations in DNA repair genes in metastatic prostate cancer ^32^. PARP inhibition has demonstrated anti-tumor activity in cancer ^33^. In a phase 2 trial (TOPARP), the PARP inhibitor olaparib demonstrated good responses in patients with mCRPC ^34^. Of 49 evaluable patients who had prior systemic therapy for CRPC, 16 (33%) responded to therapy with olaparib and 14 (88%) out of 16 responders had mutations in DNA repair genes. PARP inhibitors therefore may play a significant future therapeutic role in a subset of men with DNA repair defects ^34^. A recent phase 2 study in mCRPC demonstrated that the combination of olaparib and abiraterone had improved radiographic progression-free survival compared with abiraterone plus placebo (13.8 versus 8.2 months, *p* = 0.034) ^35^. Cytotoxic T lymphocyte-associated antigen-4 (CTLA-4) is a co-inhibitory receptor expressed on T cells and blocks T-cell activation by binding to co-stimulatory molecules ^36^. Ipilimumab is a monoclonal antibody that blocks CTLA-4 and therefore enhances anti-tumor activity by T-cell activation ^36^. Kwon *et al*. conducted a phase 3 RCT of ipilimumab versus placebo in 799 men with mCRPC and demonstrated a non-significant difference in OS of 10 months for placebo and 11.2 for the ipilimumab arm (HR 0.85, 95% CI 0.72 to 1.00, *p* = 0.053) ^37^. The only notable OS benefit was limited to a subset of patients with good prognostic features (alkaline phosphatase concentration of less than 1.5 times upper normal limit, no anemia, and no visceral metastases) ^37^.

## Conclusions

There have been significant recent strides in the management of advanced prostate cancer. Major changes in the treatment of hormone-sensitive disease have occurred on the basis of level 1 evidence to support upfront use of docetaxel plus ADT and in addition the use of androgen annihilation with abiraterone acetate plus prednisone in combination with ADT. Also, in the M0 CRPC state, there are now two randomized trials demonstrating improved MFS with the addition of apalutamide or enzalutamide in combination with ADT for patients at high risk for metastases. PARP inhibitors and immunotherapeutic agents such as CTLA-4 inhibitors are also being studied and may become a part of the treatment armamentarium in the near future.
